# Supplementary material for: A grass-specific cellulose–xylan interaction dominates in sorghum secondary cell walls
Source: Nat Commun. 2020 Nov 27;11:6081. doi: 10.1038/s41467-020-19837-z (PMC7695714; doi:10.1038/s41467-020-19837-z)
Supplement: Supplementary file 1 — Supplementary Information [file 41467_2020_19837_MOESM1_ESM.pdf]

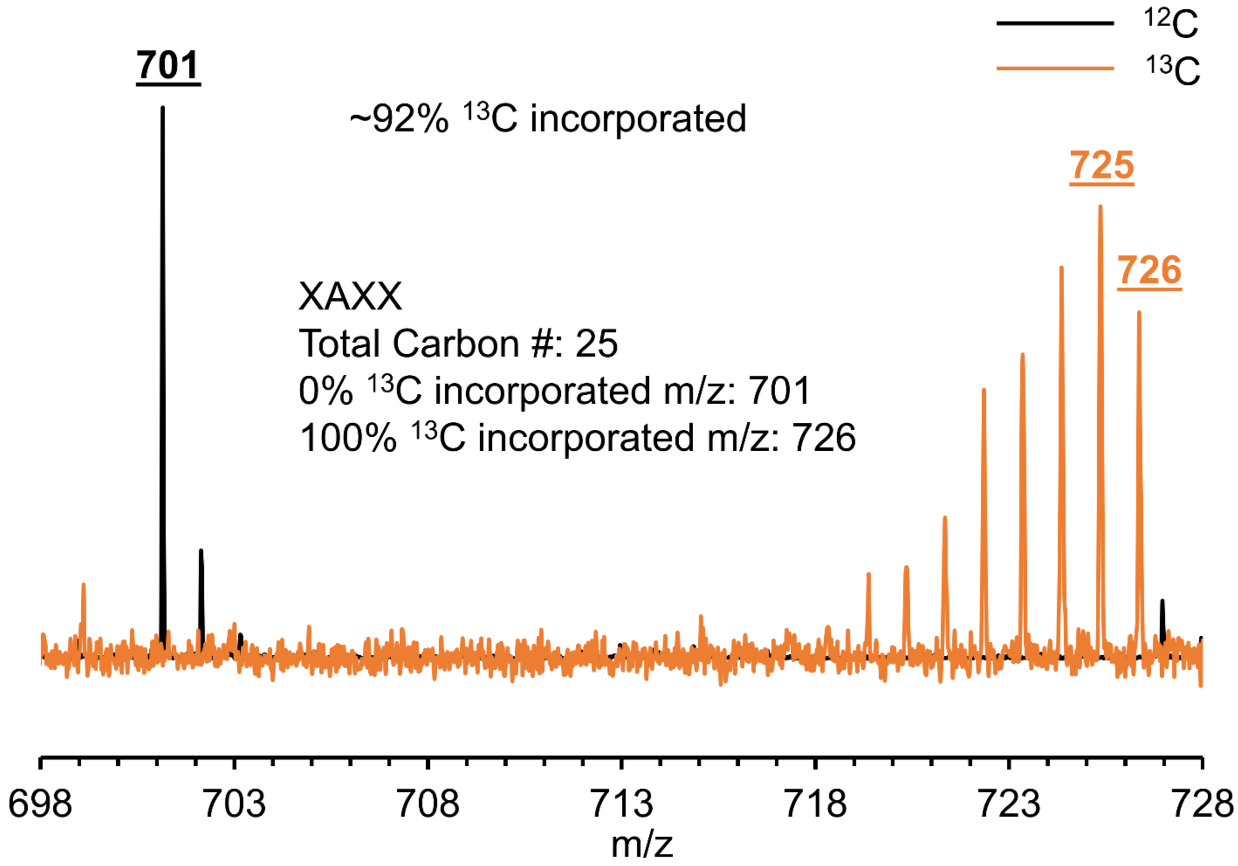

Figure S1. MALDI-TOF mass spectra of ^{12}C- and ^{13}C-oligosaccharides, XAXX, which were generated by digestion of alkali extracted xylan from sorghum secondary cell wall with GH11 xylanase. Source data are provided as a Source Data file.

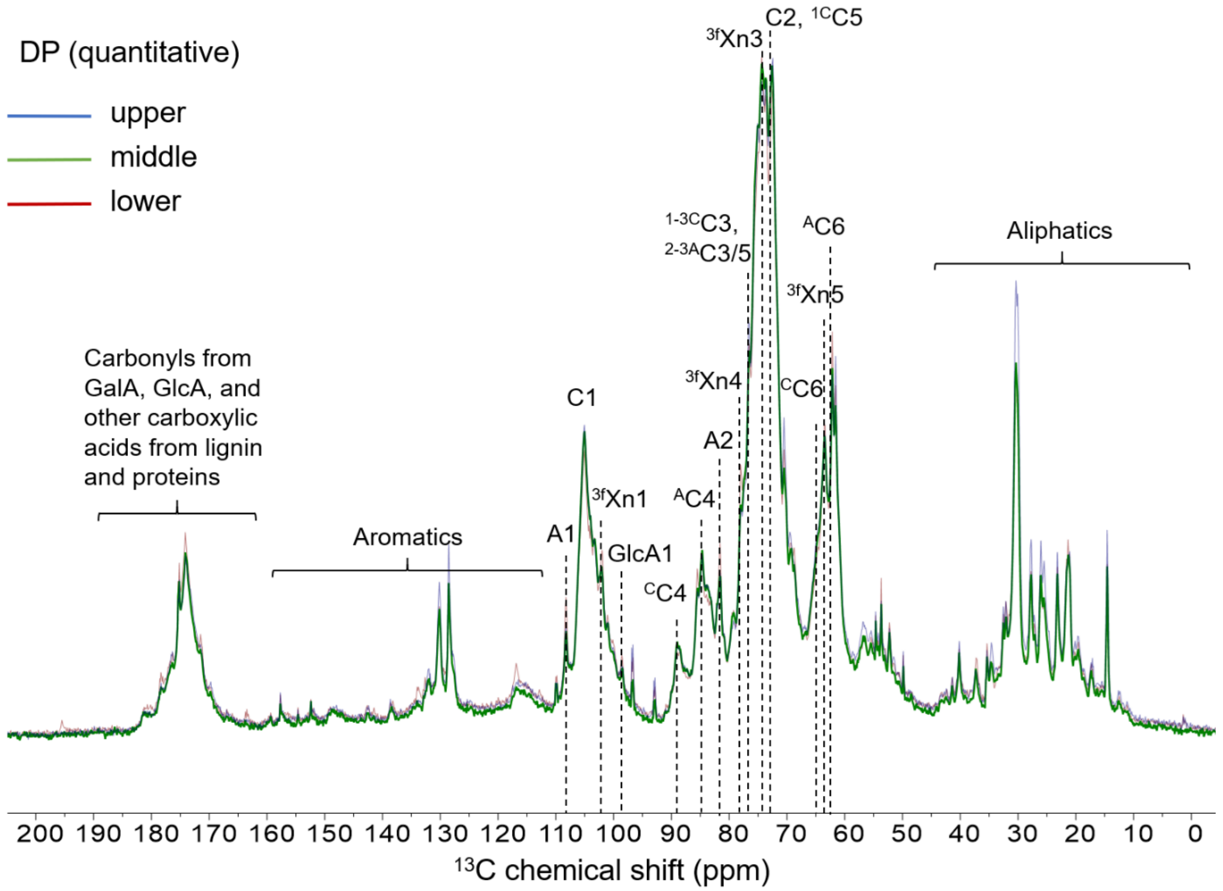

Figure S2. 1D ^{13}C quantitative DP experiments on upper, middle and lower sorghum stem internode. A recycle delay of 30 s was used to guarantee quantitative analysis. No major compositional difference was observed between different parts of stem tissue from internodes.

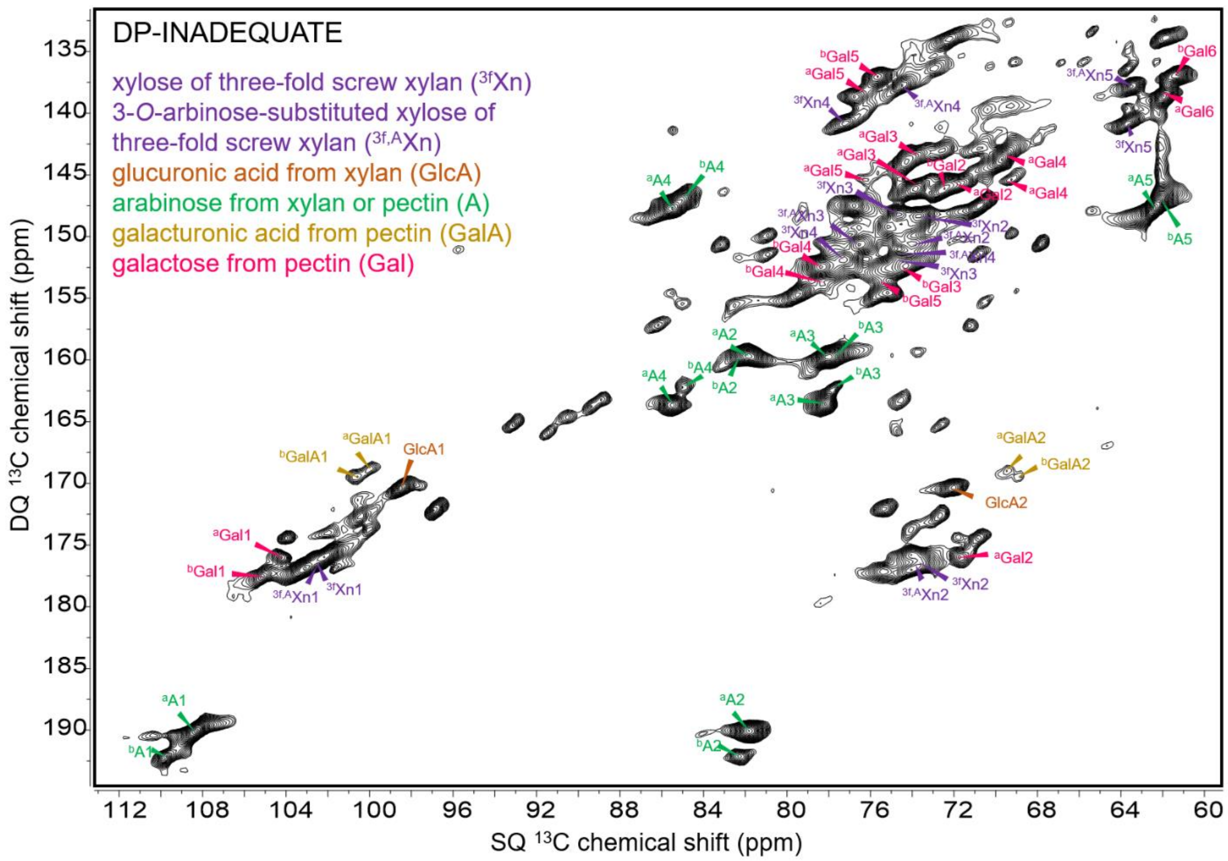

Figure S3. Mobile polysaccharides detected by refocused ^{13}C DP-INADEQUATE experiments with recycle delay of 2 s in sorghum secondary cell walls.

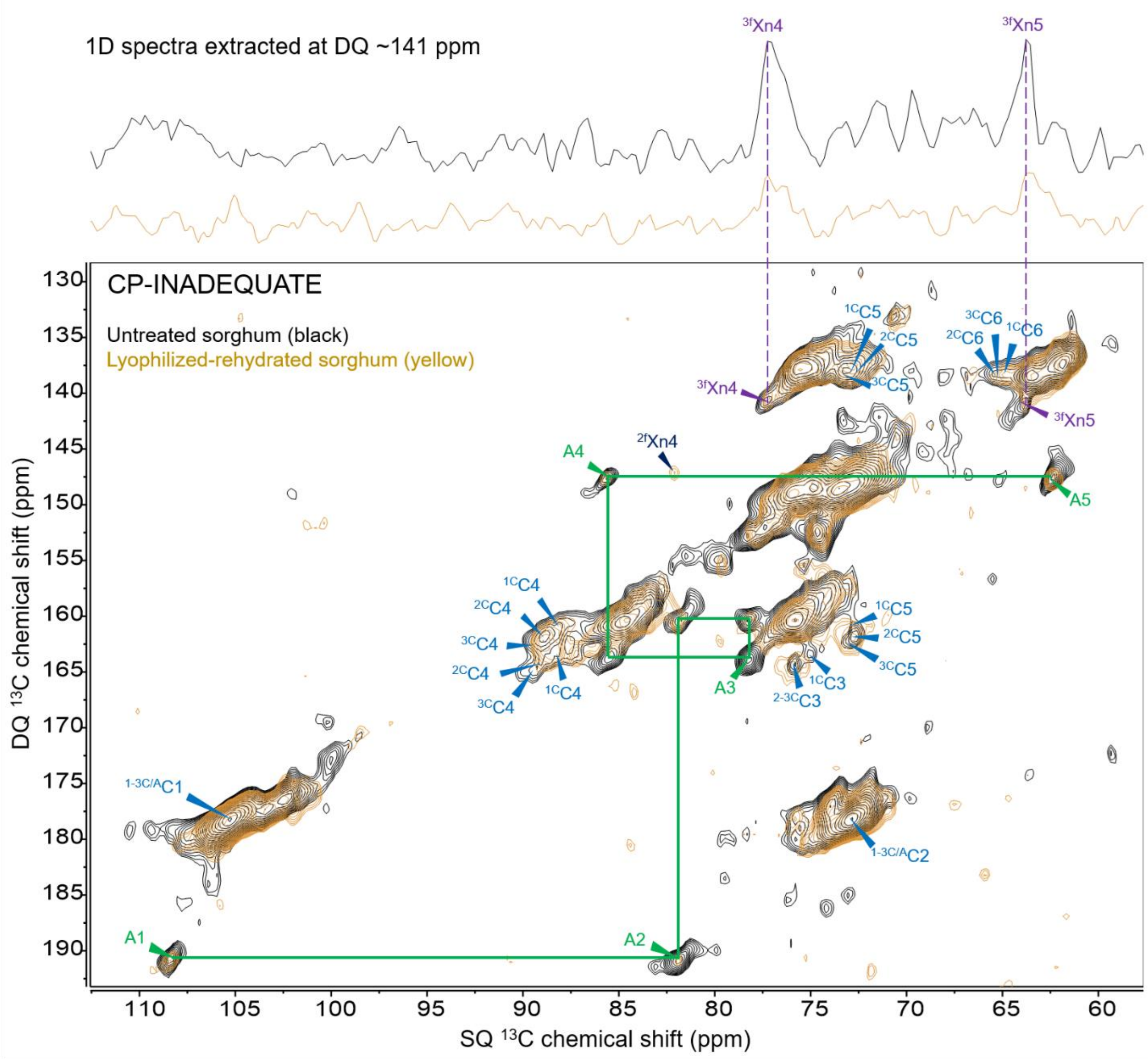

Figure S4. Spectral overlay of refocused ^{13}C CP-INADEQUATE experiments on native untreated (black) and lyophilized-rehydrated (yellow) sorghum stem tissue. The intensities of arabinose signals (i.e., A1-A5) and xylose signals from three-fold screw xylan (e.g., ^{3f}Xn4 and ^{3f}Xn5) in the lyophilized-rehydrated sample were significantly decreased compared to the untreated sample. Weak signals from xylosyl units of two-fold screw xylan (e.g., ^{2f}Xn4) were detected and the intensities of glucose signals from cellulose were enhanced in the lyophilized-rehydrated sample.

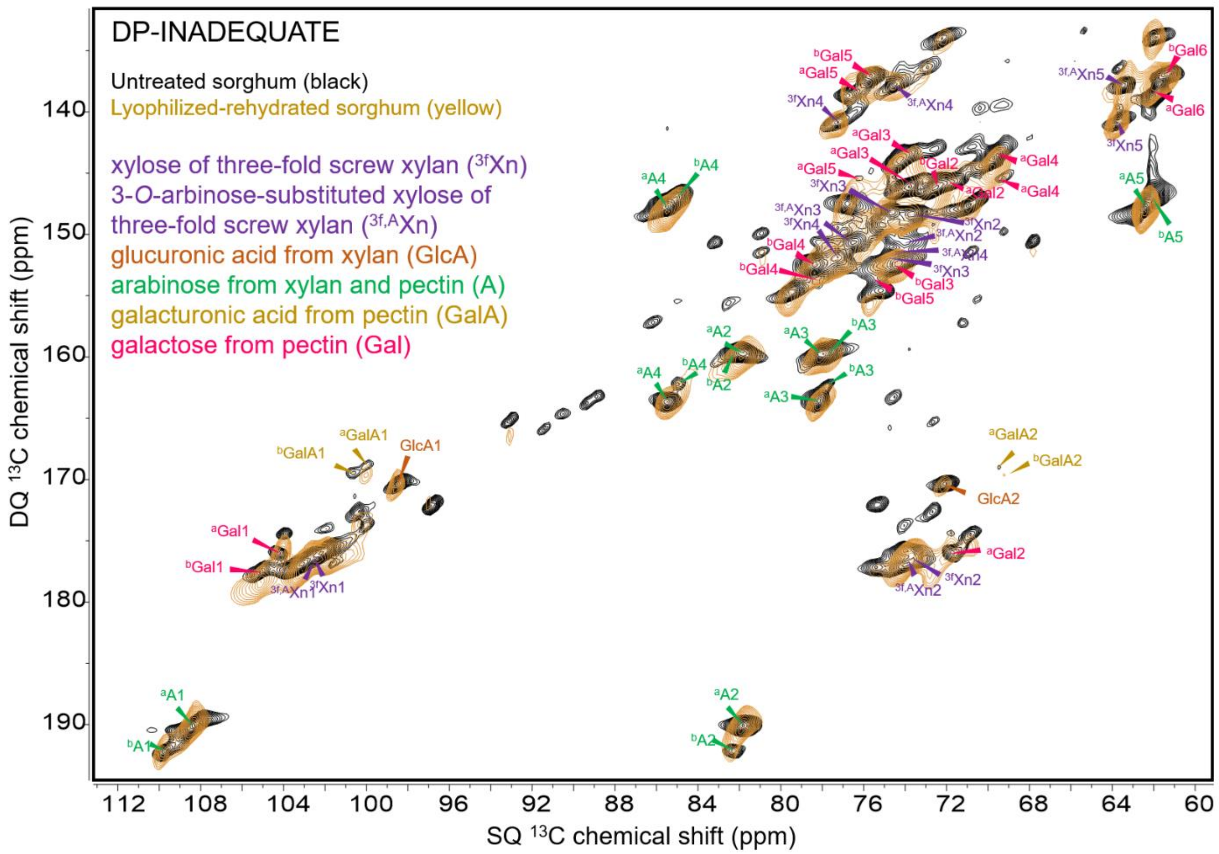

Figure S5. Spectral overlay of refocused ^{13}C DP-INADEQUATE experiments on native untreated (black) and lyophilized-rehydrated (yellow) sorghum stem tissue. In contrast to the ^{13}C DP-INADEQUATE experiments (Figure S4), the intensities of arabinose signals (i.e., A1-A5) and xylose signals from three-fold screw xylan (e.g., ^{3f}Xn4 and ^{3f}Xn5) in the lyophilized-rehydrated sample were significantly increased compared to the untreated sample.

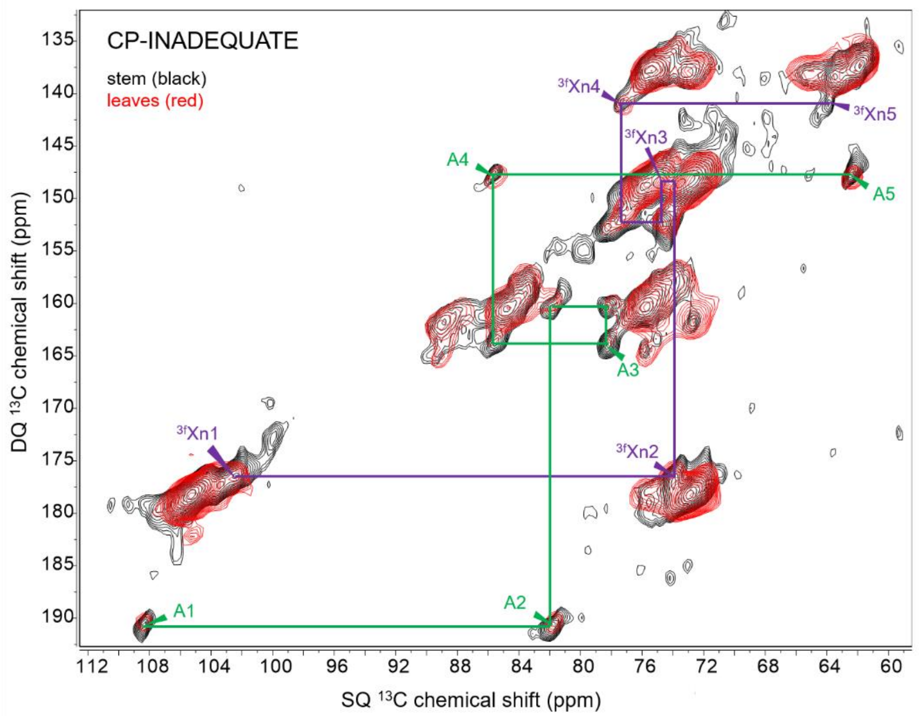

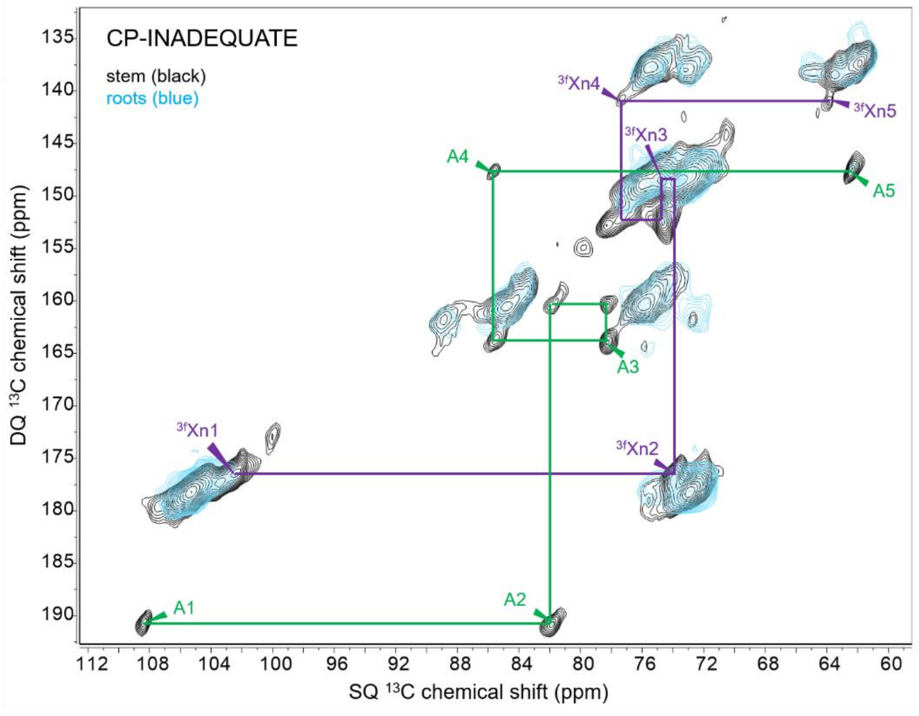

Figure S6. Spectral overlay of refocused ^{13}C CP-INADEQUATE experiments to compare sorghum leaf tissue (top panel) and root tissue (bottom panel) with stem tissue. Both sorghum leaf and root tissue show a lack of signals from two-fold screw xylan, but enhanced signals from cellulose, as in the stem tissue. Sorghum root tissue also shows a lack of signals from arabinosyl and xylosyl units from immobile three-fold screw xylan.

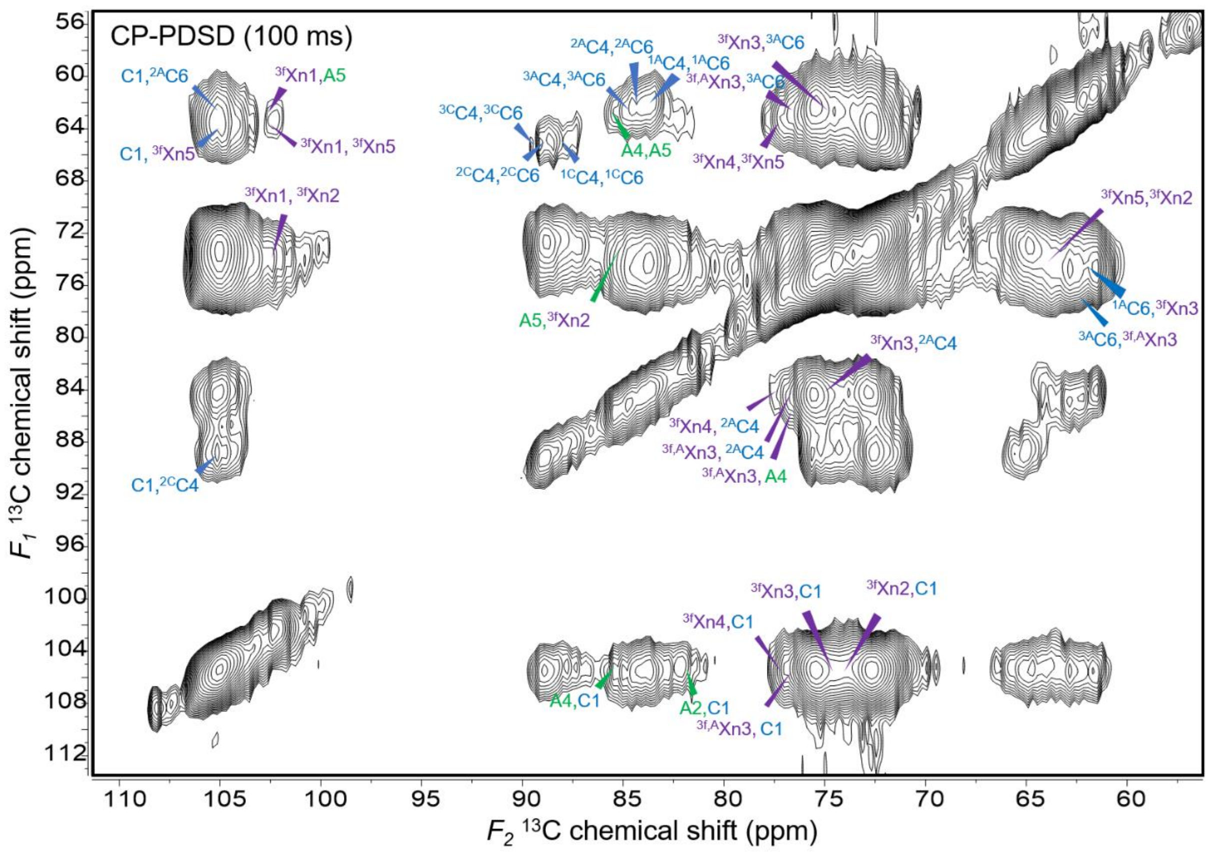

Figure S7. Close proximities between moieties from three-fold screw xylan and amorphous cellulose were indicated in sorghum secondary cell walls by CP-PDSD experiments with a mixing time of 100 ms. Similar to the CP-PDSD spectrum with short mixing time (30 ms), cross peaks that indicate interactions between arabinosyl and xylosyl units from three-fold screw xylan and glucosyl units from amorphous cellulose were detected with enhanced intensities and labelled in the spectrum.

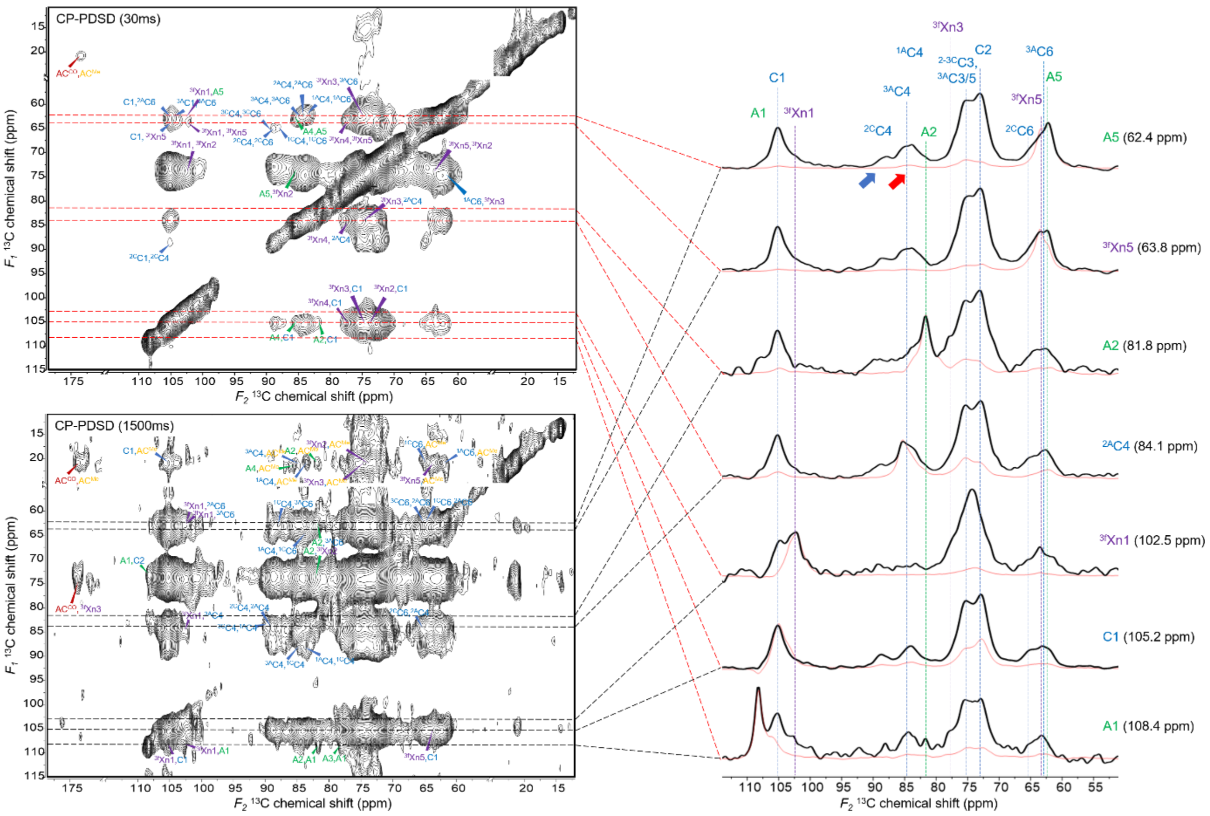

Figure S8. CP-PDSD spectra with short (30 ms) and long (1500 ms) mixing times of sorghum secondary cell walls. 1D spectra were extracted from the *F1* planes of both CP-PDSD spectra at chemical shifts, 108.4 ppm, 105.2 ppm, 102.5 ppm, 84.1 ppm, 81.8 ppm, 63.8 ppm and 62.4 ppm and demonstrated on the right panel. The blue and red arrows in the extracted 1D spectra point the chemical shift regions of C4s from crystalline and amorphous cellulose respectively, which are indicating that the moieties from xylan (A1, ^{3f}Xn1, A2, ^{3f}Xn5, and A5) interact with amorphous cellulose, but not with crystalline cellulose in close proximities.

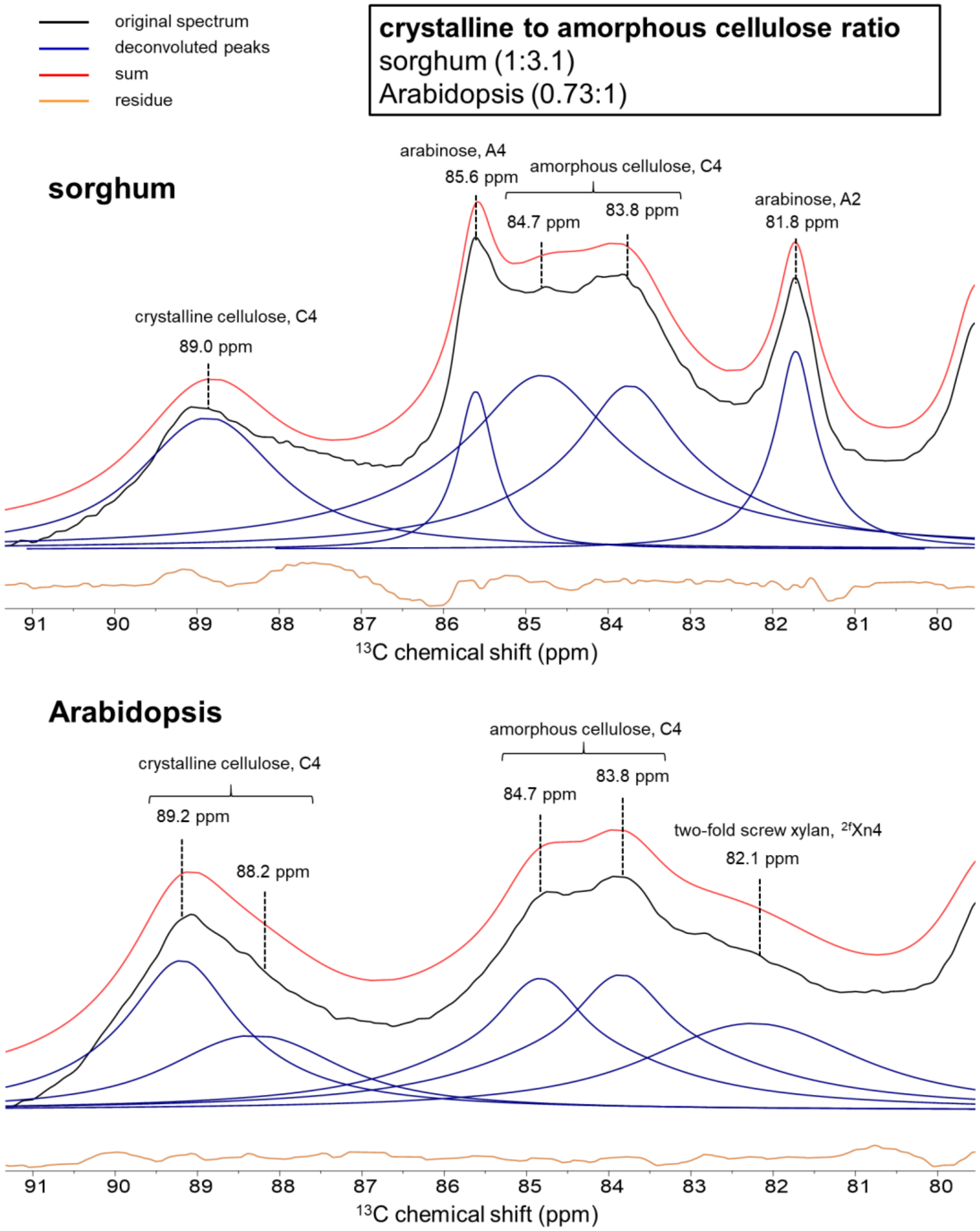

Figure S9. 1D ^{13}C CP experiments on the stem tissue of sorghum and Arabidopsis (spectra were zoomed at cellulose C4 region, 90-80 ppm). Spectra were deconvoluted and integrated by a built-in Global Spectral Deconvolution (GSD) method in MestReNova NMR processing software (Version 14.1.0). The crystalline to amorphous cellulose ratio is determined as 1:3.1 for sorghum and 0.73:1 for Arabidopsis, which is consistent with CP-PDSD determined results.

Table S1. Monosaccharide composition of the non-cellulosic components of sorghum stem internode cell walls. N.D., not detected.

	Non-cellulosic components, mol %		
**Monosaccharide**	**Upper**	**Middle**	**Lower**
Fucose	N.D.	N.D.	N.D.
Rhamnose	N.D.	N.D.	N.D.
Arabinose	12.0	12.4	12.7
Galactose	3.8	4.0	5.3
Glucose	16.6	15.8	19.2
Xylose	64.3	64.4	58.8
Mannose	N.D.	N.D.	N.D.
Galacturonic acid	2.6	2.4	2.9
Glucuronic acid	0.7	0.9	1.1

Table S2. Chemical shift assignments of polysaccharides in native sorghum stem internodes.

Polysaccharide		Chemical shift (ppm)						
**Name**	**Type, unit (denotation)**	**C1**	**C2**	**C3**	**C4**	**C5**	**C6**	**References/notes**
Cellulose	Crystalline, glucose (^{1C}C)	105.2	72.8	75.1	88.1	72.8	64.8	[1–4]
	Crystalline, glucose (^{2C}C)	105.2	72.8	75.8	89	72.6	65.4	[1–4]
	Crystalline, glucose (^{3C}C)	105.2	72.8	75.8	89.5	73	65.3	[1–4]
	Amorphous, glucose (^{1A}C)	105.2	72.8	75.1	83.8	75.1	61.8	[1–4]
	Amorphous, glucose (^{2A}C)	105.2	72.8	75.4	84.1	75.4	62.2	[1–4]
	Amorphous, glucose (^{3A}C)	105.2	72.8	75.8	84.7	75.8	62.5	[1–4]
Xylan	Two-fold screw, xylose (^{2f}Xn)	105.1	72.3	75.3	82.1	64.1	N/A	[1–3]
	Three-fold screw, xylose (^{3f}Xn)	102.5	73.7	74.7	77.3	63.8	N/A	[1,2]
	Three-fold screw, 3-*O*-arbinose-substituted xylose (^{3f,A}Xn)	102.5	74	76.7	74.6	63.6	N/A	[4,5]
	Arabinose (A)	108.4	81.8	78.2	85.6	62.4	N/A	[1,4,6]
	Glucuronic acid (GlcA)	98.6	72.4	-	-	-	-	[6]
	Acetate (AC^{CO}, AC^{Me})	174	21.4	N/A	N/A	N/A	N/A	[2]

Table S3. Quantitative ^{13}C spin-lattice relaxation time (T_1) measurements of polysaccharides in sorghum stem cell walls. Data was fitted using a biexponential function, $I(t) = A\left(1 - 2e^{-\frac{t}{T_{1A}}}\right) + B\left(1 - 2e^{-\frac{t}{T_{1B}}}\right), where\ A = 1 - B.$

	Chemical shift (ppm)	Fraction of short T_1	Short T_1	Fraction of long T_1	Long T_1
C1	105.2	0.199 ± 0.01	0 ± 0.002	0.862 ± 0.009	9.151 ± 0.235
^{1C}C6	64.8	0.194 ±0.021	0.01 ± 0.007	0.825 ± 0.017	7.864 ± 0.468
^{2C}C6	65.4	0.183 ±0.021	0.004 ± 0.005	0.88 ± 0.017	8.808 ± 0.486
^{1A}C3/5	75.1	0.208 ± 0.031	0.035 ± 0.016	0.734 ± 0.024	5.118 ± 0.491
^{1A}C6	61.8	0.535 ± 0.04	0.193 ± 0.036	0.394 ± 0.034	4.089 ± 0.771
^{2A}C6	62.2	0.51 ± 0.038	0.189 ± 0.036	0.419 ± 0.033	4.459 ± 0.784
^{3f}Xn2	73.7	0.222 ± 0.037	0.054 ± 0.028	0.682 ± 0.031	3.133 ± 0.373
^{3f}Xn3	74.7	0.218 ± 0.034	0.043 ± 0.022	0.705 ± 0.027	3.937 ± 0.428
^{3f}Xn4	77.3	0.24 ± 0.036	0.036 ± 0.02	0.664 ± 0.03	2.531 ± 0.292
^{3f}Xn5	63.8	0.382 ± 0.037	0.145 ± 0.036	0.545 ± 0.031	4.312 ± 0.597
A2	81.8	0.317 ± 0.039	0.045 ± 0.024	0.582 ± 0.032	3.048 ± 0.452
A4	85.6	0.445 ± 0.052	0.148 ± 0.045	0.505 ± 0.043	4.412 ± 0.92

Supplementary References

1. Terrett, O. M. *et al.* Molecular architecture of softwood revealed by solid-state NMR. *Nat. Commun.* **10,** 4978 (2019).
2. Simmons, T. J. *et al.* Folding of xylan onto cellulose fibrils in plant cell walls revealed by solid-state NMR. *Nat. Commun.* **7,** 13902 (2016).
3. Kang, X. *et al.* Lignin-polysaccharide interactions in plant secondary cell walls revealed by solid-state NMR. *Nat. Commun.* **10,** 347 (2019).
4. Wang, T., Salazar, A., Zabotina, O. A. & Hong, M. Structure and dynamics of Brachypodium primary cell wall polysaccharides from two-dimensional (13)C solid-state nuclear magnetic resonance spectroscopy. *Biochemistry* **53,** 2840–2854 (2014).
5. Komatsu, T. & Kikuchi, J. Comprehensive signal assignment of 13C-labeled lignocellulose using multidimensional solution NMR and 13C chemical shift comparison with solid-state NMR. *Anal. Chem.* **85,** 8857–8865 (2013).
6. Dick-Pérez, M. *et al.* Structure and interactions of plant cell-wall polysaccharides by two- and three-dimensional magic-angle-spinning solid-state NMR. *Biochemistry* **50,** 989–1000 (2011).
