# Supplementary material for: A grass-specific cellulose–xylan interaction dominates in sorghum secondary cell walls
Source: Nat Commun. 2020 Nov 27;11:6081. doi: 10.1038/s41467-020-19837-z (PMC7695714; doi:10.1038/s41467-020-19837-z)
Supplement: Supplementary file 3 — Reporting Summary [file 41467_2020_19837_MOESM3_ESM.pdf]

nature research

Corresponding author(s): Jennifer C. Mortimer

Last updated by author(s): Oct 20, 2020

Reporting Summary

Nature Research wishes to improve the reproducibility of the work that we publish. This form provides structure for consistency and transparency in reporting. For further information on Nature Research policies, see our Editorial Policies and the Editorial Policy Checklist.

Statistics

For all statistical analyses, confirm that the following items are present in the figure legend, table legend, main text, or Methods section.

n/a	Confirmed	
☐	☒	The exact sample size (*n*) for each experimental group/condition, given as a discrete number and unit of measurement
☐	☒	A statement on whether measurements were taken from distinct samples or whether the same sample was measured repeatedly
☒	☐	The statistical test(s) used AND whether they are one- or two-sided<br>*Only common tests should be described solely by name; describe more complex techniques in the Methods section.*
☒	☐	A description of all covariates tested
☒	☐	A description of any assumptions or corrections, such as tests of normality and adjustment for multiple comparisons
☒	☐	A full description of the statistical parameters including central tendency (e.g. means) or other basic estimates (e.g. regression coefficient) AND variation (e.g. standard deviation) or associated estimates of uncertainty (e.g. confidence intervals)
☒	☐	For null hypothesis testing, the test statistic (e.g. F, t, r) with confidence intervals, effect sizes, degrees of freedom and P value noted<br>*Give P values as exact values whenever suitable.*
☒	☐	For Bayesian analysis, information on the choice of priors and Markov chain Monte Carlo settings
☒	☐	For hierarchical and complex designs, identification of the appropriate level for tests and full reporting of outcomes
☒	☐	Estimates of effect sizes (e.g. Cohen's d, Pearson's r), indicating how they were calculated

Our web collection on statistics for biologists contains articles on many of the points above.

Software and code

Policy information about availability of computer code

Data collection	All NMR data on stem internodes samples and Arabidopsis stem sample were collected by using VNMRJ v4.2A, NMR data on root and leaf samples were collected by using Bruker Topspin v2.1
Data analysis	All NMR data were analyzed by MestReNova v14.0, T1 data were fitting by using OriginPro 2016

For manuscripts utilizing custom algorithms or software that are central to the research but not yet described in published literature, software must be made available to editors and reviewers. We strongly encourage code deposition in a community repository (e.g. GitHub). See the Nature Research guidelines for submitting code & software for further information.

Data

Policy information about availability of data

All manuscripts must include a data availability statement. This statement should provide the following information, where applicable:

- Accession codes, unique identifiers, or web links for publicly available datasets
- A list of figures that have associated raw data
- A description of any restrictions on data availability

All relevant data supporting the findings of this manuscript is available within the paper and its supplementary information files. All the unprocessed NMR data files are available at https://doi.org/10.25582/DATA.2020-09.2061884/1661467. The Source Data underlying Supplementary Fig. S1 are provided in a Source Data file.

Field-specific reporting

Please select the one below that is the best fit for your research. If you are not sure, read the appropriate sections before making your selection.

- [x] Life sciences
- [ ] Behavioural & social sciences
- [ ] Ecological, evolutionary & environmental sciences

For a reference copy of the document with all sections, see nature.com/documents/nr-reporting-summary-flat.pdf

Life sciences study design

All studies must disclose on these points even when the disclosure is negative.

Sample size	No sample size calculation was performed. Tissue from 2 plants grown side-by-side (due to 13CO2 costs and chamber size, it is not feasible to grow more) were chopped into 1-2 mm pieces and well mixed to form a pool. All experiments were performed at least three times on a newly packed sample each time, taken from the pool to represent the whole and avoid bias measurement.
Data exclusions	No exclusions
Replication	Tissue from 2 plants grown side-by-side were chopped into 1-2 mm pieces and well mixed to form a pool. All experiments were performed at least three times on a newly packed sample each time, taken from the pool. All attempts at replication were successful.
Randomization	No need - all plants were of identical genotypes.
Blinding	Blinding was not used. The NMR data was collected by two authors (at two remote sites), and analyzed by a third author. This is an observation of material (wild type sorghum), not a comparison between variables.

Reporting for specific materials, systems and methods

We require information from authors about some types of materials, experimental systems and methods used in many studies. Here, indicate whether each material, system or method listed is relevant to your study. If you are not sure if a list item applies to your research, read the appropriate section before selecting a response.

Materials & experimental systems

n/a	Involved in the study	
☒	☐	Antibodies
☒	☐	Eukaryotic cell lines
☒	☐	Palaeontology and archaeology
☒	☐	Animals and other organisms
☒	☐	Human research participants
☒	☐	Clinical data
☒	☐	Dual use research of concern

Methods

n/a	Involved in the study	
☒	☐	ChIP-seq
☒	☐	Flow cytometry
☒	☐	MRI-based neuroimaging
